# Multiple sclerosis under the age of ten: the challenge of a rare diagnosis in a special population – a case series

**DOI:** 10.3389/fnins.2023.1297171

**Published:** 2023-12-20

**Authors:** Inês V. Carvalho, Constança Soares dos Santos, Joana Amaral, Joana Afonso Ribeiro, Cristina Pereira, Rui Pedro Pais, Filipe Palavra

**Affiliations:** ^1^Center for Child Development – Neuropediatrics Unit, Hospital Pediátrico, Centro Hospitalar e Universitário de Coimbra, Coimbra, Portugal; ^2^Neurology Department, Centro Hospitalar e Universitário de Coimbra, Coimbra, Portugal; ^3^Medical Image Department – Neuroradiology Unit, Centro Hospitalar e Universitário de Coimbra, Coimbra, Portugal; ^4^Laboratory of Pharmacology and Experimental Therapeutics, Coimbra Institute for Clinical and Biomedical Research (iCBR), Faculty of Medicine, University of Coimbra, Coimbra, Portugal; ^5^Clinical Academic Center of Coimbra, Coimbra, Portugal

**Keywords:** multiple sclerosis, paediatric, children, diagnosis, treatment

## Abstract

**Introduction:**

Multiple sclerosis (MS) is a chronic inflammatory demyelinating and degenerative disease of the central nervous system which, when it begins before the age of 18, is defined as paediatric MS. Most common clinical presentations include long tract involvement, brainstem/cerebellum syndromes, optic neuritis and acute disseminated encephalomyelitis. Paediatric-onset MS typically has a more inflammatory-active course and a higher lesion burden in imaging studies, but an extensive post-relapse recovery, with a slower long-term disability progression. The first demyelinating clinical attack occurs before 10 years old in less than 1% of patients, and, in this special population, the condition has particularities in clinical presentation, differential diagnosis, diagnostic assessment, current treatment options and outcome.

**Clinical cases:**

We present the cases of four Caucasian children (2 girls) diagnosed with relapsing–remitting MS before the age of ten, with a mean age at the time of the first relapse of 7.4 ± 2.4 years. Clinical presentation included optic neuritis, myelitis, brainstem syndrome, and acute disseminated encephalomyelitis. Baseline MRI identified several lesions, frequently large and ill-defined. Two patients were included in clinical trials and two patients remain in clinical and imaging surveillance.

**Conclusion:**

Diagnosis of MS before the age of 10 years is rare, but it has significant long-term physical and cognitive consequences, as well as a substantial impact on the current and future quality of life of the child and family. Early and correct diagnosis is essential. Prospective, randomized, large cohort studies are needed to assess the efficacy and safety of disease-modifying treatments in children under the age of ten.

## 1 Introduction

Multiple sclerosis (MS) is a chronic inflammatory demyelinating and degenerative disease that affects the central nervous system (CNS) (Brola and Steinborn, 2020; Jakimovski et al., 2022). When the first demyelinating event occurs before the age of 18, it is called paediatric-onset MS and this can actually occur in 3–5% of cases (Brola and Steinborn, 2020; Ramphul et al., 2020; Luca et al., 2021), according to the literature, with some studies reporting a rising incidence of MS in children (Brenton et al., 2020; Margoni et al., 2021). Seventeen percent of patients diagnosed at paediatric age had their first relapse before the age of 10 (Deiva, 2020), corresponding to 0.2 to 0.7% of all MS patients (Ruggieri et al., 2004). In children younger than 10 years old, MS is as frequent in girls as in boys and female predominance is established only during puberty (Ruggieri et al., 2004; Langille et al., 2019; Deiva, 2020). Despite fundamental similarities, paediatric MS has distinct features, especially in pre-pubertal children, which can complicate the process of differential diagnosis (Ruggieri et al., 2004).

Children can present with a wide variety of manifestations (Shaw and Alvord, 1987; Maeda et al., 1989; Giroud et al., 1990; Cole et al., 1995; Ruggieri et al., 1999; Ghezzi et al., 2004; de Albuquerque et al., 2012; Sivaraman and Moodley, 2016; Alroughani and Boyko, 2018; Öztürk et al., 2018). The commonest clinical presentations in paediatric MS are long tract involvement, brainstem/cerebellum syndromes, optic neuritis and acute disseminated encephalomyelitis (ADEM). In children under ten, the occurrence of infection or vaccination in the month preceding the first attack tends to be more common. Ataxia, pure motor signs and polysymptomatic presentations are also more frequent (Ruggieri et al., 2004; Mikaeloff et al., 2006; Huppke et al., 2014; Bduignan et al., 2019; Deiva, 2020).

The diagnosis of MS requires evidence of CNS demyelination with dissemination in time (DIT) and space (DIS) (Bduignan et al., 2019). It is particularly challenging and frequently delayed in younger children (Ruggieri et al., 2004). The McDonald Criteria 2017 (Thompson et al., 2018) have a sensitivity of 71% and a specificity of 95% when applied to the paediatric population, but they should be considered with caution in children younger than 12 years (Fadda et al., 2018). In pre-pubertal children, new relapses (Luca et al., 2021) or the identification of new lesions on control MRI are the preferred criteria for corroborating DIT. According to the International Paediatric Multiple Sclerosis Study Group (2013 definitions), the first attack can be an ADEM; a diagnosis of MS in a child with ADEM requires at least one additional non-ADEM episode and fulfilment of DIT and DIS criteria (Krupp et al., 2013). Paraclinical evidence further supports DIT, but it is worth emphasizing that oligoclonal bands are less commonly found in children under 10 years old (Ruggieri et al., 2004; Mikaeloff et al., 2006; Deiva, 2020).

In clinical practice, it can be quite challenging to differentiate MS from other demyelinating syndromes that can occur in children, including Neuromyelitis Optica Spectrum Disorder (NMOSD), Myelin Oligodendrocyte Glycoprotein Antibody-associated Disease (MOGAD), ADEM and monophasic disorders such as optic neuritis and transverse myelitis (Alroughani and Boyko, 2018; Brenton, 2022; Jakimovski et al., 2022). Other diseases can mimic it, such as infectious encephalitis or meningoencephalitis, inflammatory vasculopathies and hereditary metabolic disorders (Alroughani and Boyko, 2018; Bduignan et al., 2019).

When compared to adult-onset disease, paediatric-onset MS typically has a more inflammatory-active course with more frequent relapses (Gorman et al., 2009; Alroughani and Boyko, 2018; Jakimovski et al., 2022) and a higher MRI lesion burden (Fragoso, 2018; Deiva, 2020), but an extensive post-relapse recovery (sometimes complete) after the initial attack can occur, with a slower long-term disability progression (Harding et al., 2013; Jakimovski et al., 2022; Nicotera et al., 2022). However, cerebral atrophy may be present from the beginning of the disease (Deiva, 2020). Additionally, early attacks result in impaired brain development (Bonacchi et al., 2021) and poorer cognitive performance, with long-term consequences (Nicotera et al., 2022). In children with MS, the intense inflammatory process that occurs early in the disease course highlights the necessity of timely initiation of efficacious therapies, in order to reduce disease activity and disability progression (Nicotera et al., 2022). However, children younger than 12 years start disease-modifying treatment (DMT) less often and newer agents are used to lesser extent (Krysko et al., 2018). Currently, only three DMTs (fingolimod, teriflunomide and dimethyl fumarate) have been tested in phase III trials; fingolimod and teriflunomide are approved for children older than 10 years old and dimethyl fumarate for children over 12 (Nicotera et al., 2022). Use of first-line injectable is based on clinical trials conducted in adults, observational studies and personal experience (Margoni et al., 2021). High-efficiency therapies are used off-label in patients with very active disease who did not respond to first-line medications. Several clinical trials are currently studying different DMTs in paediatric patients, but many protocols exclude younger children, particularly under the age of 10 (Fadda et al., 2021; Margoni et al., 2021; Nicotera et al., 2022).

With the aim of increasing global knowledge about this entity that manifests so early in life, we report a case series of four patients diagnosed with MS established in our centre (Hospital Pediátrico, Centro Hospitalar e Universitário de Coimbra), before the age of 10, highlighting the peculiarities of clinical presentation, differential diagnosis, clinical assessment, and current challenges to prescribing treatment for these children, which has an impact on their prognosis. Follow-up duration varied between twelve months and almost four years.

## 2 Case descriptions

### 2.1 Case 1

A Caucasian boy aged 4 years and 10 months developed gait difficulty with weakness and instability associated with lower limb dysesthesias. The symptoms progressed over a couple of weeks and then started to slowly improve. His past medical history was irrelevant, except for the diagnosis of SARS-CoV-2 and Varicella Zoster Virus infection 1 month before symptom’s onset. There was no family history of degenerative or neurological disorders.

Neurological examination revealed a spastic asymmetric tetraparesis with generalized hyperreflexia, bilateral extensor plantar responses and gait ataxia. Remaining physical examination was unremarkable.

**Figure 1:**
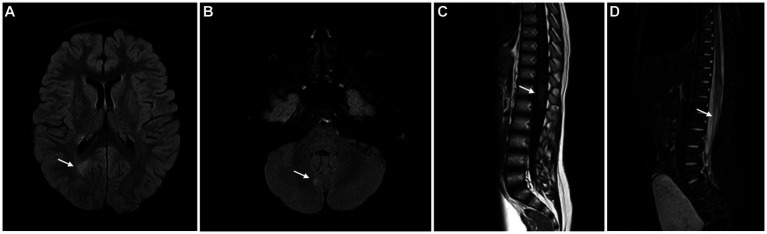
Brain MRI showing FLAIR/STIR hyperintense lesions – periventricular white matter **(A)**, right cerebellar hemisphere **(B)** and conus medullaris **(C)**, compatible with demyelinating lesions. The lesion in the conus medullaris shows enhancement after gadolinium administration **(D)**.

Brain and spinal cord Magnetic Resonance Imaging (MRI) (Figure 1) showed multiple T2 hyperintense lesions in the right parieto-occipital transition, right anterior peri-atrial region, left parietal juxtacortical region, right cerebellar hemisphere, left middle cerebellar peduncle middle, dorsal medulla, and *conus medullaris* (D12-L1), compatible with demyelinating lesions. Gadolinium enhancement was evident in a lesion localized at the *conus medullaris*.

Blood laboratory exams included the search for Myelin Oligodendrocyte Glycoprotein (MOG) and Aquaporin-4 (AQP4) antibodies using cell-based indirect immunofluorescence, and were negative. Additionally, no 25-hydroxyvitamin D deficiency was detected. There was serological evidence of remote exposure to Epstein–Barr virus (EBV). Cerebrospinal fluid (CSF) cytochemical analysis was normal and there was no evidence of central nervous system infection. Oligoclonal bands were detected only in the CSF, suggesting intrathecal synthesis.

After a multidisciplinary discussion, the diagnosis of MS was assumed, based on the presence of one clinical syndrome (myelitis) and imaging evidence for DIS (with spinal, juxtacortical, infratentorial and periventricular lesions) and DIT (with enhancing and non-enhancing lesions). The presence of oligoclonal bands provided additional evidence of DIT.

The patient was started on pulse corticosteroid therapy with intravenous methylprednisolone 30 mg/kg/day during 5 days. Clinical response to corticoid therapy was good, with reduction of Expanded Disability Status Scale (EDSS) score from 3.0 to 2.0. The boy resumed all previous activities.

Clinical and imaging surveillance was instituted and DMT was not initiated, after a long discussion with the family, so that the consent process was fully informed. After 12 months of follow-up, the child is clinically stable without relapses or incapacity progression. Follow-up MRI performed 12 months after the initial event did not reveal new T2 hyperintense lesions, and many of the previously identified ones were attenuated.

### 2.2 Case 2

A 5-years and 10 months old previously healthy Caucasian boy was brought to the paediatric emergency department due to a five-day history of diplopia and irritability without headache, fever, or seizures. The boy was diagnosed with an upper respiratory infection a week before. Remaining past medical and family history were inconspicuous. On examination, the left eye was hypertropic with right cephalic tilt. Fundoscopy was normal and there were no sensory or motor deficits, ataxia or meningeal signs.

**Figure 2:**
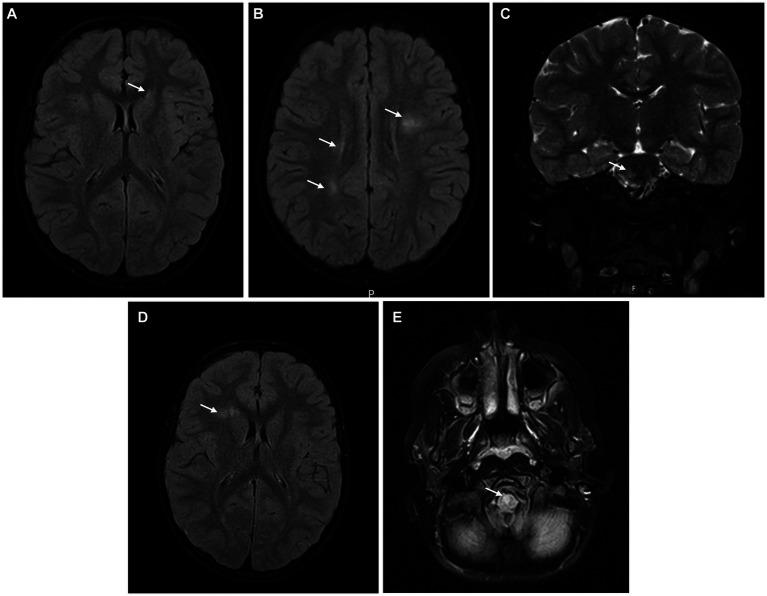
Brain MRI at symptom’s onset **(A–C)** showing several T2 hyperintense lesions in the periventricular white matter, radiate crown, and right pons, without gadolinium-enhancement; after 6 months **(D)**, a small new non-enhancing T2 hyperintense lesion in the right frontal periventricular region was observed; at 12 months **(E)**, a new T2 hyperintense lesion in the anterior bulbocervical transition was detected.

Blood laboratory exams including complete blood count, liver enzymes, kidney function and inflammatory markers were normal. Serum MOG and AQP4 antibodies were not detected and there was evidence of previous non-recent Epstein–Barr virus (EBV) infection. CSF analysis revealed pleocytosis (100 leukocytes/mm^3^), hyperproteinorraquia (78.7 mg/dL) and normal glucose levels. Oligoclonal bands were absent in the serum and CSF. Brain MRI (Figures 2A–C) revealed several T2 hyperintense lesions in the periventricular white matter, radiate crown and right pons without gadolinium enhancement and so a diagnosis of ADEM was considered. The patient completed 5 days of high-dose intravenous methylprednisolone followed by an oral steroid taper for 6 weeks with a complete resolution of the symptoms.

Brain MRI (Figure 2D) was repeated 6 months after symptoms’ onset, and it revealed a small new non-enhancing T2 hyperintense lesion in the right frontal periventricular region. Spinal MRI showed a small T2 hyperintense lesion in the *conus medullaris* without contrast enhancement. The patient was maintained under clinical and imaging surveillance, but 12 months after the diagnosis of ADEM the boy developed subacute right hemiparesis; a new T2 hyperintense lesion was evident in the anterior bulbocervical transition (Figure 2E). The patient met the 2017 McDonald’s criteria for MS and started on pulse corticosteroid therapy with methylprednisolone during 5 days, with complete recovery of the motor symptoms. Immunomodulatory treatment was proposed to the family, but they refused, after a multidisciplinary discussion with the clinical team, preferring to adopt a more assertive clinical and radiological surveillance approach. After 4 years of follow-up, the patient remains stable without new or enlarging T2 hyperintense lesions, relapses, or disability progression (EDSS 1.0). DMT has not been started so far, but the family knows that its introduction will not be delayed if additional disease activity is identified.

### 2.3 Case 3

A Caucasian girl aged 9 years and 4 months reported acute decrease in visual acuity of the right eye without headache or ocular pain. When specifically questioned, the girl remembered a previous episode of lower limb paraesthesia with spontaneous resolution. Remaining past medical and familial history were not relevant. Visual acuity was 20/20 on the left eye, but she was only able to count fingers with her right eye. A relative afferent pupillary defect (RAPD) in the right eye and a nasal and superior central visual field loss were evident. There was no inflammation in the anterior chamber or vitreous on slit lamp examination. Fundus examination was normal in the left eye, but mild oedema of the disc was observed in the right eye. The patient was hospitalized with the diagnosis of unilateral optic neuritis. There was no evidence of additional neurological deficits.

**Figure 3:**
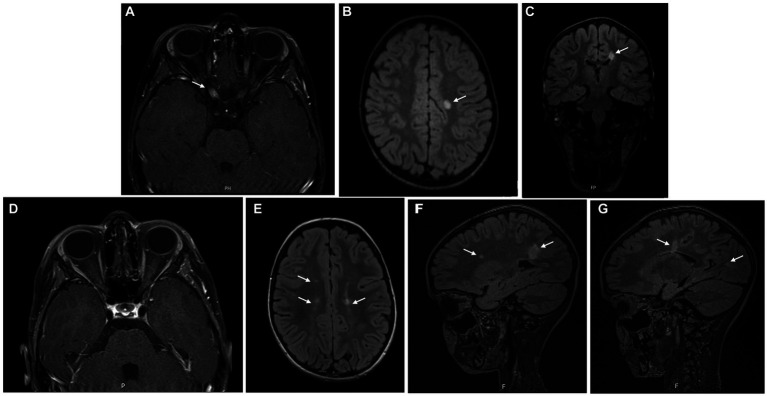
Brain MRI at symptom’s onset **(A–C)** showing slight thickening of the pre-chiasmatic segment of the right optic nerve, with contrast enhancement **(A)**, as well as T2 hyperintense periventricular and subcortical lesions **(B,C)**; 12 months later **(D–G)**, resolution of the right optic nerve thickening and contrast-enhancement was observed, but several new T2 hyperintense lesions were identified.

Blood tests were normal except for mild 25-hydroxyvitamin D deficiency. Serum MOG and AQP4 antibodies were not detected. CSF analysis revealed pleocytosis (20 lymphocytes/mm^3^) with normal protein and glucose count. Eight oligoclonal bands were detected exclusively in the CSF. Brain and orbital MRI (Figures 3A–C) revealed slight thickening of the pre-chiasmatic segment of the right optic nerve with contrast enhancement, as well as T2 hyperintense periventricular and subcortical lesions.

A seven-day course of pulse corticosteroid therapy was prescribed with significant improvement. Visual acuity continued to improve over the next months, with complete recovery. Brain MRI was repeated 12 months later (Figures 3D–G) revealing new periventricular and juxtacortical T2 hyperintense lesions, some with enhancement after administration of intravenous gadolinium. The girl was diagnosed with MS based on the presence of one clinical syndrome (optic neuritis) and imaging evidence for DIS and DIT. The identification of oligoclonal bands corroborated DIT. After completing 10 years old, she started treatment with pegylated interferon beta-1a, as part of an open-label clinical trial.

### 2.4 Case 4

A 9-years and 7 months old previously healthy Caucasian girl was referred to the neuropaediatrics department due to subacute onset of horizontal binocular diplopia. Neurological examination revealed left abducens nerve paralysis, discrete pyramidal signs on the left upper and lower limbs, as well as discrete left lower limb appendicular ataxia. Blood laboratory exams, including serum MOG and AQP4 antibodies, were unremarkable. CSF chemical and cytological analyses were normal. Thirteen oligoclonal bands were detected only in the CSF.

**Figure 4:**
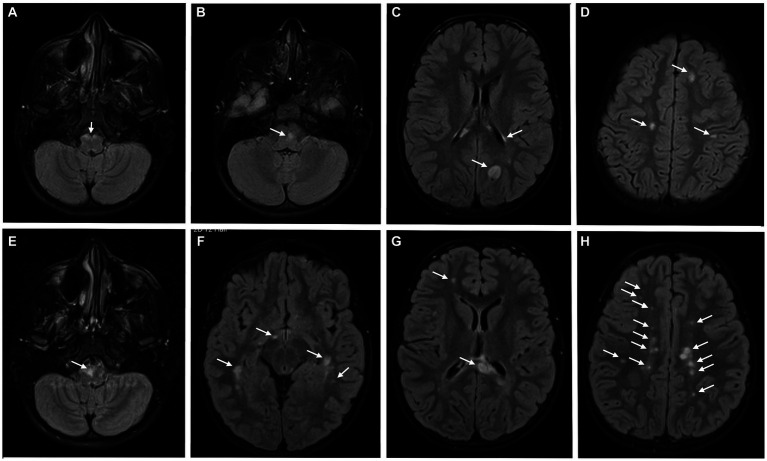
Brain MRI at the diagnosis **(A–D)**, revealing several T2 hyperintense periventricular and infratentorial lesions; 6 months after the first relapse **(E–H)**, a significant increase in the number and volume of T2 hyperintense lesions was detected.

Brain MRI (Figures 4A–D) revealed several T2 hyperintense periventricular and infratentorial lesions, one of them being located close to the emergence of the left abducens nerve, which also showed enhancement after administration of intravenous gadolinium, in an incomplete ring pattern. There were also T1 hypointense periventricular and infratentorial lesions.

The girl was diagnosed with MS based on the presence of one clinical syndrome (brainstem syndrome) and imaging evidence for DIS and DIT, which was also corroborated by the presence of oligoclonal bands. Based on the diagnosis, the patient was started on pulse corticosteroid therapy with methylprednisolone during 5 days, with complete resolution of the diplopia.

She was maintained under clinical and imaging surveillance without DMT, after a first discussion of this topic with the family. Despite the absence of new symptoms, brain MRI was repeated 6 months after the first relapse (Figures 4E–H) with a significant increase in lesions burden, now including multiple periventricular, subcortical and infratentorial lesions, some with pseudotumor characteristics. Some previously identified T2 hyperintense lesions were less evident or even absent. Having turned 10 years old, she was included in a clinical trial comparing fingolimod, siponimod and ofatumumab.

Table 1 summarizes the clinical and imaging characteristics of this series of 4 children diagnosed with MS before reaching 10 years of age.

**Table 1:** Summary of patients’ characteristics.

Patient	Sex	Race	Age at clinical presentation (years)	Clinical presentation	Baseline MRI	CSF (cytology and biochemistry)	Oligoclonal bands	MOG and AQP4 antibodies	Age at diagnosis (years)	Clinical evolution	Control MRI	Follow-up duration (years)	DMT
1	Male	Caucasian	4	Myelitis	T2 hyperintense spinal, juxtacortical, infratentorial and periventricular lesions; enhancing and non-enhancing lesions	Normal	Several bands (not present in serum)	Negative	4	No relapses, no disability progression, EDSS 2.0	(12 months) No new T2 hyperintense lesions	1	–
2	Male	Caucasian	5	ADEM	T2 hyperintense infratentorial and periventricular lesions; only non-enhancing lesions	Pleocytosis (100 leukocytes/mm^3^); elevated protein (78.7 mg/dL) and normal glucose levels	Absent	Negative	6	One relapse (brainstem syndrome), EDSS 1.0	(6 months) New T2 hyperintense periventricular and infratentorial lesions	4	–
3	Female	Caucasian	9	Unilateral optic neuritis	Thickening and contrast enhancement of the optic nerve; T2 hyperintense non-enhancing periventricular and subcortical lesions	Pleocytosis (20 lymphocytes/mm^3^), normal protein and glucose levels	8 bands (not present in serum)	Negative	10	No relapses, no disability progression, EDSS 0	(12 months) New T2 hyperintense periventricular and juxtacortical lesions	4	Clinical trial (open-label pegylated interferon beta-1a)
4	Female	Caucasian	9	Brainstem syndrome	T2 hyperintense and T1 hypointense periventricular and infratentorial lesions; enhancing and non-enhancing lesions	Normal	13 bands (not present in serum)	Negative	9	No relapses, no disability progression, EDSS 1.0	(6 months) New T2 hyperintense periventricular, subcortical and infratentorial lesions	2	Clinical trial (fingolimod *vs* siponimod *vs* ofatumumab)^*^

^*^Disease-modifying treatment was started after completing 10 years old. AQP4, Aquaporin-4; CSF: cerebrospinal fluid; DMT, disease-modifying treatment; EDSS, Expanded Disability Status Scale; MOG, Myelin Oligodendrocyte Glycoprotein; MRI, Magnetic Resonance Imaging.

## 3 Discussion

We presented four examples of Caucasian children diagnosed with MS, with symptoms starting between 4 and 9 years of age, 50% of whom (*n* = 2) being girls. In children under 10, the prevalence is similar between girls and boys, while in older children and adults a female predominance is usually observed. Hormonal interference during puberty may explain this difference (Langille et al., 2019; Brenton et al., 2020; Deiva, 2020). All the children we have just described are of Caucasian race, but this situation may result from the racial distribution of Portuguese population. These are all the cases we observed with MS under 10 years, between January 2016 and December 2022, which corroborates the rarity of this clinical situation in this age group (hormonal influences may also justify the increase of incidence during and after puberty).

Children who develop MS are influenced by the same genetic and environmental risk factors as adults (Bduignan et al., 2019; Brola and Steinborn, 2020; Fisher et al., 2020). In our cohort, testing for genes associated with increased risk of developing the disease, including haplotypes of human leukocyte antigen (HLA), was not performed. Two patients underwent EBV serology, both with evidence of previous infection, most probably asymptomatic (the other 2 patients were referred from other hospitals, and we did not have this data at the time of diagnosis). A history of EBV infection is associated with a significant increase in the risk of developing MS in both children and adults (Brola and Steinborn, 2020; Soldan and Lieberman, 2023). Present or personal history of obesity, head trauma and family history of CNS demyelinating disorders or systemic autoimmune diseases were absent in all patients. One presented vitamin D deficiency, but the role of this variable in very early disease is not entirely clear; nevertheless, the association with increased susceptibility to MS and higher rate of relapses has been reported (Mowry et al., 2010; Banwell et al., 2011; Bduignan et al., 2019). Exposure to parental smoking, a previously reported risk factor (Mikaeloff et al., 2007; Brenton et al., 2020), was not evaluated in our cohort.

Our patients developed four different clinical presentations, portraying the diversity of MS symptoms. Except for ADEM, clinical syndromes did not differ from the classical pictures described in adolescents and adults, which are myelitis, optical neuritis and brainstem syndrome. As previously reported in the literature, motor symptoms and ADEM occurred in the younger patients (cases 1 and 2) and multifocal symptoms were observed in some of them. Despite more frequent is younger children (Ruggieri et al., 2004), seizures were not reported in our cohort and other paroxysmal phenomena, rare in this age group (Ruggieri et al., 2004), were indeed absent. Prodromic symptoms were not reported. Retrospectively, only one of our patients reported symptoms (lower limb paraesthesia) attributable to MS prior to the first identified relapse. Diagnostic delay in case 1 evidenced the challenge of MS diagnosis in younger children, a situation that was worsened by the fact that the family was initially living in another country, where access to healthcare is considered more complex. It is worth to mention that comparison with previous reports of MS before the age of 10 need to be done with caution, because many case reports were published more than one decade ago (before the generalized search for MOG and AQP4 antibodies in these patients), at a time when the diagnostic criteria would certainly not be the same we use today.

Coming back to our patients, baseline MRI identified several lesions, frequently large and ill-defined. Compared to adults, children tend to demonstrate a higher lesion load and volume on MRI, with more active and infratentorial lesions; some may even assume pseudotumor characteristics (Langille et al., 2019; Brenton et al., 2020). T1 hypointense lesions were identified in one patient. Despite less often in paediatric populations, these lesions increase diagnostic specificity for MS.

Ancillary laboratory data, especially CSF testing, were included in the diagnostic flowchart of all our cases. CSF findings were diverse, from normal cell count to mild lymphocytosis and marked neutrophilic pleocytosis, which is rare in adolescents and adults with MS, but can occur in younger children. These differences further increase the challenge of MS diagnosis in this special population. Despite previous evidence that oligoclonal bands are less commonly found in children under 10 years old (Mikaeloff et al., 2006; Deiva, 2020), they were identified in 3 of our 4 patients.

Differential diagnosis should be particularly careful in younger children. Inclusion of other demyelinating conditions in differential diagnosis schemes, particularly ADEM and MOGAD, more frequent under 10 years of age, is particularly important. Metabolic, infectious and autoimmune systemic diseases should also be considered, particularly if there are atypia or warning signs, in addition to the clinical syndromes considered classic (Brola and Steinborn, 2020). All patients developed relapsing–remitting MS as primary progressive form is extremely rare in children, and it implies the consideration of alternative diagnosis, particularly at very early ages (Boiko et al., 2002; Gorman et al., 2009; Alroughani and Boyko, 2018; Langille et al., 2019; Deiva, 2020; Fadda et al., 2021). Despite a follow-up between 12 months and almost 4 years, only one of our patients presented a second attack 12 months after the first demyelinating event. Paediatric MS is usually associated with a higher relapse rate and a shorter interval between the first and second attacks, with around 40% of children experiencing a second episode of demyelination within a year of the first presentation, 60% within 2 years, and 66% within 3 years (Ruggieri et al., 2004). Some studies report a lower annualized relapse rate (ARR) in younger children with intervals as long as 10 years cited in the literature (Ruggieri et al., 2004; Deiva, 2020).

New MRI lesions in the follow-up scan (performed 6 to 12 months after the first relapse) were identified in three of our children. The number of lesions varied between one non-acute small plaque and a significant increase in disease burden, with several enhancing and non-enhancing lesions. This illustrates that even before the age of 10, MS is a heterogeneous condition. Compared to adults, children tend to have a rapid increase in lesion burden in the first years of the disease, but in our patients complete or almost complete resolution of some previously seen T2 hyperintense lesions was also observed. Reduction of previously seen T2-hyperintense lesions is usually more extensive in children due to less axonal loss and demyelination or better remyelination mechanisms (Langille et al., 2019; Brenton et al., 2020).

During the follow-up period, which was also heterogeneous in our cohort, for the reasons already mentioned, the patients maintained low EDSS scores (Kurtzke, 1983), corroborating the known slow progression of physical disability in the initial stages of paediatric MS (Mikaeloff et al., 2004; Nicotera et al., 2022). However, despite taking approximately 10 years longer for patients with paediatric MS to reach irreversible disability, they reach these landmarks at a biological age around 10 years younger than patients with adult-onset disease (Renoux et al., 2007, 2008; Alroughani and Boyko, 2018; Fragoso, 2018). Extremely early-onset MS appears to be associated with an unfavourable functional outcome (Ruggieri et al., 2004).

All patients reported good academic results, without changes in school programs, despite the diagnosis. Nevertheless, cognitive impairment has not been studied with formal tests in our population, to date. Cognitive impairment occurs in 30–75% of children with MS, and younger age of symptoms’ onset correlates with lower intelligence quotient (Banwell and Anderson, 2005; Amato et al., 2014; Cardoso et al., 2015; Lysenko et al., 2023).

All patients were treated with corticosteroid pulses, with a complete or almost complete remission of symptoms, due to the high plasticity of the CNS in children. Previous reports indicate a complete recovery from the first attack in 80% of children younger than 10 years old and a shorter mean time of recovery than adults (Ruggieri et al., 2004). Milder disability after the first clinical episode in the very young child (<6 years) has been described (Huppke et al., 2014). However, this recovery can also be misleading when assessing disease severity (Hacohen and Eshaghi, 2021).

As currently there are no DMTs approved for children under the age of 10, two patients were included in on-going clinical trials after this age and two patients remain in clinical and imaging surveillance. This decision was naturally shared with the family, which plays a critical role in managing the therapeutic approach to this disease. The number of clinical trials and approved DMTs for MS in adults are increasing each year. Therapeutic options for children, particularly under the age of 10, are not following this tendency. The reduced number of patients with paediatric-onset MS and the ethical issues associated with inclusion of children in clinical trials limit the development of new therapeutic options. In addition, the introduction of DMTs in children involves new challenges such as the acceptance and adherence to injectable DMTs, the risk of potentially severe adverse reactions and the impact in the developing immune system, growth, cognition, and socialization. In the absence of clinical trials, defining the correct dose (dependent on child’s weight and age) can also be a challenge. It is hard to evaluate the benefits and risks of a long-term immunosuppressant therapy at such a young age, and families greatly value (from our practice-based perspective) issues related to safety, at an early stage of the disease.

## 4 Conclusion

Diagnosis of MS before the age of 10 years old is rare, but it may have significant long-term physical and cognitive consequences, as well as a substantial impact on current and future quality of life of the child and family. Early and correct diagnosis is essential. Prospective, randomized, large cohort studies are needed to assess the efficacy and safety of DMTs in this special population, among paediatric MS. The impact of DMTs in a developing immune system and long-term effects of such a therapy, that will be maintained for decades, in principle, generate some concerns in clinical practice, which often dictate, in the therapeutic decision-making process, the adoption of a more expectant attitude, at least until the moment when there are drugs with formal approval by regulatory authorities to be used with evidence. This is a rather orphan area of knowledge, but it can be very important, in the future, for understanding the biology of demyelination/remyelination in early MS, which could result in the eventual development of new treatment strategies for the disease.

## Data availability statement

The original contributions presented in the study are included in the article/supplementary material, further inquiries can be directed to the corresponding author.

## Ethics statement

Written informed consent was obtained from the minor(s)' legal guardian/next of kin for the publication of any potentially identifiable images or data included in this article.

## Author contributions

IC: Conceptualization, Investigation, Writing – original draft. CS: Conceptualization, Writing – review & editing. JA: Conceptualization, Writing – review & editing. JR: Conceptualization, Writing – review & editing. CP: Conceptualization, Writing – review & editing. RP: Conceptualization, Writing – review & editing. FP: Conceptualization, Writing – original draft, Writing – review & editing.
